# Ablation of Central Serotonergic Neurons Decreased REM Sleep and Attenuated Arousal Response

**DOI:** 10.3389/fnins.2018.00535

**Published:** 2018-08-07

**Authors:** Kanako Iwasaki, Haruna Komiya, Miyo Kakizaki, Chika Miyoshi, Manabu Abe, Kenji Sakimura, Hiromasa Funato, Masashi Yanagisawa

**Affiliations:** ^1^International Institute for Integrative Sleep Medicine (WPI-IIIS), University of Tsukuba, Tsukuba, Japan; ^2^Department of Cellular Neurobiology, Brain Research Institute, Niigata University, Niigata, Japan; ^3^Department of Anatomy, Faculty of Medicine, Toho University, Tokyo, Japan; ^4^Department of Molecular Genetics, University of Texas Southwestern Medical Center, Dallas, TX, United States; ^5^Life Science Center, Tsukuba Advanced Research Alliance, University of Tsukuba, Tsukuba, Japan

**Keywords:** 5-HT neuron, serotonin, sleep, REMS, diphtheria toxin, brain, mouse model

## Abstract

Sleep/wake behavior is regulated by distinct groups of neurons, such as dopaminergic, noradrenergic, and orexinergic neurons. Although monoaminergic neurons are usually considered to be wake-promoting, the role of serotonergic neurons in sleep/wake behavior remains inconclusive because of the effect of serotonin (5-HT)-deficiency on brain development and the compensation for inborn 5-HT deficiency by other sleep/wake-regulating neurons. Here, we performed selective ablation of central 5-HT neurons in the newly developed *Rosa-diphtheria toxin receptor (DTR)-tdTomato* mouse line that was crossed with *Pet1*^*Cre*/+^ mice to examine the role of 5-HT neurons in the sleep/wake behavior of adult mice. Intracerebroventricular administration of diphtheria toxin completely ablated tdTomato-positive cells in *Pet1*^*Cre*/+^*; Rosa-DTR-tdTomato* mice. Electroencephalogram/electromyogram-based sleep/wake analysis demonstrated that central 5-HT neuron ablation in adult mice decreased the time spent in rapid eye movement (REM) sleep, which was associated with fewer transitions from non-REM (NREM) sleep to REM sleep than in control mice. Central 5-HT neuron-ablated mice showed attenuated wake response to a novel environment and increased theta power during wakefulness compared to control mice. The current findings indicated that adult 5-HT neurons work to support wakefulness and regulate REM sleep time through a biased transition from NREM sleep to REM sleep.

## Introduction

Sleep/wake behavior is regulated by distinct groups of neurons located from the forebrain to the medulla (Luppi et al., 2011; Weber and Dan, 2016; Scammell et al., 2017). Among a variety of neuronal groups, monoaminergic neurons, such as dopaminergic and noradrenergic neurons, are considered to be wake-promoting neurons (Carter et al., 2010; Eban-Rothschild et al., 2016). Although serotonin (5-HT)-containing neurons are also regarded as wake-promoting neurons (Saper et al., 2005; Scammell et al., 2017), the role of serotonergic neurons on sleep/wake behavior remains inconclusive (Ursin, 2002; Monti, 2011).

In early studies conducted by Jouvet et al. dorsal raphe lesions decreased sleep time in correlation with a reduction in brain serotonin (Jouvet, 1999). The administration of p-chlorophenylalanine (PCPA), which is an irreversible inhibitor of tryptophan hydroxylase (TPH), the rate-limiting enzyme in the synthesis of 5-HT, induced insomnia, suggesting a role of the ascending serotonergic system in sleep enhancement (Mouret et al., 1968; Jouvet, 1999). However, PCPA also block TPH1 and reduced peripheral 5-HT, which is involved in a variety of functions(El-Merahbi et al., 2015). Furthermore, PCPA reduces dopamine and noradrenalin levels in the brain(Dailly et al., 2006) which may alter sleep/wakefulness. Hypothermia after PCPA treatment may also lead to insomnia (Murray et al., 2015).

Subsequently, unit recordings of raphe nucleus neurons revealed that their activity was usually high during wakefulness, low during NREM sleep and almost absent during REM sleep (McGinty and Harper, 1976; Trulson and Jacobs, 1979; Jacobs and Fornal, 1999). Pharmacological studies targeting 5-HT receptors supported the wake-promoting effects of 5-HT signaling. Systemic administration of a 5-HT1A agonist (Monti and Jantos, 2003), 5-HT1B agonist (Bjorvatn and Ursin, 1994; Monti et al., 1995) and 5-HT2 agonist (Dugovic et al., 1989) increased wakefulness and decreased NREM sleep and REM sleep. Intracerebroventricular infusion of a 5-HT3 agonist also increased wakefulness and decreased NREM sleep and REM sleep (Ponzoni et al., 1993). Among the 14 5-HT receptors in mice, 5-HT1A, 1B, 2A, 2C, and 7 have been investigated through the examination of sleep/wake behavior in mice deficient in these receptors (Hannon and Hoyer, 2008). The time spent in wakefulness was greater in *5-HT2A*- and *2C*-deficient mice than in control mice (Frank et al., 2002; Popa et al., 2005) and was not altered in *5-HT1A*- or *5-HT1B*-deficient mice (Boutrel et al., 1999, 2002), which is seemingly inconsistent with the wake-promoting effect of agonists for 5-HT1A, 1B, and 2A/C. *5-HT7*-deficient mice also had a normal total wake time (Hedlund et al., 2005).

Since 5-HT receptors are diverse in their regional and subcellular distribution and differ in their effect on intracellular signaling and neuron activity (Hannon and Hoyer, 2008), the role of the entire 5-HT neuron network in sleep/wake behavior cannot be characterized by investigating mice that are deficient in each 5-HT receptor but must be assessed by examining mice that are deficient in 5-HT or 5-HT neurons. Mice deficient in Tph2, a rate-limiting enzyme for brain 5-HT, showed normal time spent in wakefulness, NREM sleep and REM sleep with a longer NREM sleep duration than control mice (Solarewicz et al., 2015). The unexpectedly mild sleep phenotype of *Tph2*-deficient mice could be due to a compensation for the inborn deficiency by other sleep/wake-regulating neurons. In addition, loss of serotonin signaling during brain development modulates the response of developing thalamocortical axons to netrin-1 (Bonnin et al., 2007) and results in disturbances in the formation of neural circuits related to sleep/wake behavior, potentially masking the role of 5-HT in sleep/wake behaviors of wild-type mice. In fact, acute disruption of Tph2 in the adult raphe nuclei led to a reduction in time spent in behavioral sleep (Whitney et al., 2016).

In addition to their effect on wakefulness, 5-HT neurons have been proposed to play an inhibitory role in the switch from NREM sleep to REM sleep based on the cessation of firing of dorsal raphe neurons during REM sleep (McCarley, 2007). Consistent with this finding, microinjection of a 5-HT1A agonist (Monti and Jantos, 2003) and 5-HT2 agonist (Amici et al., 2004) into the laterodorsal tegmental nucleus, which contains REM sleep-active cholinergic neurons, decreased REM sleep. The increased REM sleep of *5-HT1A*-deficient mice (Boutrel et al., 2002) and *5-HT1B*-deficient mice (Boutrel et al., 1999) compared to that of wild-type mice furthers support the role of 5-HT1A and 1B in REM sleep suppression. *5-HT7*-deficient mice have exhibited shorter REM sleep time than wild-type mice (Hedlund et al., 2005).

In addition to 5-HT, 5-HT neurons express vesicular glutamate transporter 3 (VGLUT3), which is responsible for the uptake of glutamate into synaptic vesicles (Fu et al., 2010; Hioki et al., 2010), and 5-HT neurons release glutamate to modulate reward behavior (Liu et al., 2014). 5-HT neurons in the raphe nuclei also express some neuropeptides (Okaty et al., 2015). Thus, neurotransmitters other than 5-HT may be involved in sleep/wake regulation, and thus the sleep/wake behavior of 5-HT neuron-ablated mice may be distinct from that of mice deficient in 5-HT.

To examine the role of 5-HT neurons in sleep/wake behaviors of adult mice, the effects of 5-HT deficiency on brain development and on the maturation of sleep/wake behavior during the early postnatal stage must be excluded. Here, we performed selective ablation of central 5-HT neurons by the intracerebroventricular (ICV) administration of diphtheria toxin (DT) (Murphy, 2011) in the newly developed *Pet1*^*Cre*/+^; *Rosa-diphtheria toxin receptor (DTR)-tdTomato* mouse line to examine sleep/wake behavior based on electroencephalogram (EEG)/electromyogram (EMG). Ablation of central 5-HT neurons in adult mice resulted in a decrease in time spent in REM sleep, which was associated with fewer transitions from NREM sleep to REM sleep than in control mice, and attenuated the increase in wakefulness in response to a novel environment.

## Methods

### Animals

All of the procedures were conducted in accordance with the Guidelines for Animal Experiments of University of Tsukuba and were approved by the Institutional Animal Care and Use Committee of University of Tsukuba (Approved protocol ID # 140144). Mice were raised in our breeding colony under controlled conditions (12-h light/dark cycle, lights on at 9:00 A.M., 55 ± 5% humidity, and *ad libitum* access to water and food). Mice were weaned at 4 weeks of age and were housed in groups of four or five. Pet1-Cre mice (*B6.Cg-Tg(Fev-cre)*^1*Esd*/*J*^ (Scott et al., 2005; Deneris, 2011) were used in this study. At any stage of this study, when mice exhibited symptoms of being severely unhealthy, such as massive weight loss or an inability to walk, we sacrificed them by cervical dislocation under deep anesthesia and did not use the data associated with that animal in this study. We housed mice and performed all experiments at 23 ± 2°C.

### Production of Rosa-loxP-stop-loxP-DTR-tdTomato mice

For the generation of *Rosa26-loxP-stop-loxP-DTR-tdTomato* mice (*Rosa-DTR-tdTomato* mice), a genomic fragment that contained the ROSA locus was isolated from C57BL/6 mouse genomic BAC clone from an RP23 mouse genomic BAC library (Advanced Geno TEchs Co). The targeting vector had a splice acceptor (SA), two loxP sequences that were inserted before the repeated SV40 polyadenylation signal and after the “stop” sequence that contained the terminator of the yeast His3 gene and SV40 polyadenylation signal. A 1.7-kb fragment of the FRT-PGK-gb2-neo-FRT-loxP cassette (Gene Bridges) was inserted after the repeated SV40 polyadenylation signal. After the second loxP sequence, the targeting vector contained the coding sequence of the DTR, simian HBEGF followed by IRES and tdTomato sequences. The targeting vector was linearized and electroporated into the C57BL/6N ES cell line RENKA. Correctly targeted clones were injected into the eight-cell stage ICR mouse embryos, which were cultured to produce blastocysts and later transferred to pseudopregnant ICR females. The resulting male chimeric mice were crossed with female C57BL/6N mice to establish the LSL-DTR-tdTomato; neo line. To remove the neomycin resistance gene with the FLP-FRT system, LSL-DTR-tdTomato; neo mice were crossed with Actb-FLP knock-in mice (Kono et al., 2017). LSL-DTR-tdTomato mice were further crossed with *Pet1*^*Cre*^ mice to establish *Pet1*^*Cre*^; DTR-tdTomato mice. Gene-modified mice were regularly crossed with C57BL/6J mice (CLEA Japan) to minimizing genetic drift.

### Surgery

For the infusion of DT into the lateral cerebral ventricle, a cannula was implanted. The guide cannula was prepared by cutting stainless steel wire (Ziggy's tubes and wires, 23R304-36) into 10-mm lengths, and the dummy cannula was made by cutting a 30-gauge stainless steel tube (Ziggy's tubes and wires, #30R304-36) into a 15-mm-long tube and folding one end of the tube. An injection cannula was also made from 23- and 30-gauge stainless steel tubes.

Male mice (2.7–5.5 months old) were anesthetized with isoflurane, and the cranium was exposed. A guide cannula was inserted under stereotaxic control, which was tilted 30° to the sagittal plane to make room for the implantation of EEG electrodes. The tip of the cannula was targeted to the following coordinates: anteroposterior (AP): −0.50 mm, mediolateral (ML): 1.00 mm, and dorsoventral (DV): −2.25 mm. Immediately after cannula implantation, each mouse was implanted with an EEG/EMG electrode containing 4 electrode pins and two flexible stainless steel wires as reported previously (Komiya et al., 2018). The electrode pins were lowered to the dura under stereotaxic control, and the electrode socket was subsequently attached to the skull with dental cement (3M ESPE, Ketac Cem Aplicap). Two ipsilateral pins (AP: 0.5 mm, ML: 1.77 mm, DV: −1.3 mm; and AP: −4.5 mm, ML: 1.77 mm, DV: −1.3 mm) were used for the EEG recording. For the EMG recording, two stainless wires were inserted into the neck extensor muscles.

### ICV injection of aCSF and DT

After habituation to the recording conditions, the mice were injected with 1 μL aCSF through the guide cannula using a 5-μl syringe (Hamilton #85) under anesthesia with isoflurane. The aCSF contained 125 mM NaCl, 2.5 mM KCl, 1.25 mM NaH_2_PO_4_, 26 mM NaHCO_3_, 10 mM glucose, 2 mM CaCl_2_ and 1 mM MgCl_2_. Twelve to fourteen days after the aCSF injections, 5 ng DT was injected at a concentration of 5 ng/μL.

### EEG/EMG recording and analysis

EEG/EMG signaling was obtained and analyzed as previously described with some modifications (Funato et al., 2010). EEG/EMG signals were amplified, filtered (EEG: 0.3–300 Hz; EMG: 30–300 Hz) with a multi-channel amplifier (NIHON KODEN, #AB-611J), and digitized at a sampling rate of 250 Hz using an analog-to-digital converter (National Instruments #PCI-6220). The EEG/EMG data were visualized and semi-automatically analyzed by MATLAB-based software followed by visual inspection. Each 20-s epoch was staged into wakefulness, NREM sleep and REM sleep. Wakefulness was scored based on the presence of low amplitude, fast EEG activity and high amplitude, variable EMG activity. NREM sleep was characterized by high amplitude, delta (1–4 Hz)-frequency EEG waves and low EMG tonus, whereas REM sleep was staged based on theta (6–9 Hz)-dominant EEG oscillations and EMG atonia. EEG/EMG was subsequently recorded for 11 days after injections. The recordings on the 9th and 10th day after the injections were analyzed beginning at ZT0 (9:00 A.M.). The total time spent in wakefulness, NREM sleep, and REM sleep were derived by summing the total number of 20-s epochs in each state. Mean episode durations were determined by dividing the total time spent in each state by the number of episodes of that state. Epochs that contained movement artifacts were included in the state totals but excluded from subsequent spectral analysis. EEG signals were subjected to Fourier transform analysis from 1 to 30 Hz with 1-Hz bins using the MATLAB-based custom software. The EEG power density in each frequency bin was expressed as a percentage of the mean total EEG power over all frequency bins and sleep/wake states.

### Arousal response to a novel cage

A mouse that was housed in a home cage for at least 1 week was transferred to a novel cage that contained fresh bedding at ZT7. The sleep/wake behavior was assessed for 24 h from ZT0. To assess EEG power density during wakefulness from ZT7 to ZT12, delta and theta range power were normalized by the total EEG power over all frequency (1–30 Hz) during wakefulness from ZT7 to ZT12. We performed novel cage experiments using 8 mice, and the EEG/EMG data from 2 mice that contained movement artifacts were included in state totals but excluded from the spectral analysis.

### Blood glucose and body temperature

Blood glucose was measured from tail blood using Glutest system (Sanwa Kagaku) at the late light phase in a fed condition. The body temperature of a mouse was monitored using a digital thermometer (BDT-100, Bio Research Center) with a rectal probe (RET-3, Bio Research Center) without anesthesia and was acquired in 15 s, including mouse restraint, probe insertion into the rectum, stabilized temperature recording, and probe removal. After full acclimatization, the mice only needed to be gently restrained for the body temperature measurement.

We measured body temperature of *Pet1*^*Cre*/+^; *Rosa-DTR-tdTomato* mice at ZT5, 11, 13, and 23 on the 9th and 10th days after the aCSF injection. Subsequently, we injected DT into the same *Pet1*^*Cre*/+^; *Rosa-DTR-tdTomato* mice and measured their body temperature at ZT5, 11, 13, and 23 on the 9th and 10th days after the DT injection.

### Histological examination

Mice were deeply anesthetized with isoflurane and transcardially perfused with PBS followed by 4% paraformaldehyde (PFA) in PBS. The brains were postfixed in 4% PFA at 4°C overnight, cryoprotected in 30% sucrose in PBS for 2 days, embedded in OCT compound (Sakura Finetech), and stored at −80°C until use. The brains were cryosectioned coronally at a thickness of 40 μm and stored in tissue cryoprotectant solution at −20°C.

We used anti-5-HT antibody to visualize 5-HT neurons. More than 99% of Tph2-positive cells are positive for 5-HT and vice versa (Hioki et al., 2010). The sections were rinsed with PBS and incubated in 0.4% Block Ace (Snow Brand Milk Products) in PBS with 0.1% Tween20 (0.1% PBST) for 1–2 h at room temperature. This procedure was followed by overnight incubation with goat anti-5-HT (1:5000; ImmunoStar #20079) and rabbit anti-RFP (1:2500; MBL #PM005) antibodies in 0.2% Block Ace in 0.1%PBST overnight at 4°C. The sections were rinsed with PBS and then incubated with AlexaFluor488-conjugated donkey anti-goat IgG (1:1000; ThermoFisher #A11055) and AlexaFluor594-conjugated donkey anti-rabbit IgG (1:1000; ThermoFisher # A21207) antibodies in 0.2% Block Ace in 0.1%PBST overnight at 4°C. Sections were rinsed with PBS and mounted with Vectashield mounting medium with 4',6-diamidino-2-phenylindole (DAPI) (Vector Lab, #H-1200). The signals were visualized using a confocal microscope (Zeiss, #LSM700) with Plan-Apochromat 20x/0.8 M27 (Zeiss). AlexaFluor488 or AlexaFluor594 was excited with 488 or 555 nm laser beams, respectively, and their fluorescence was obtained 490–587 or 585- nm emission wavelength, respectively. The sections 4.7, 6.6, and 7.1 mm posterior from bregma were used for cell counting for the dorsal/median raphe, raphe pallidus and raphe obscurus, respectively. The images of stained sections were acquired using ZEN 2010 and analyzed using ZEN Black software (Zeiss). The raphe areas were shown on the display with the scale in which 1 cm on the display represents 25 μm on the section. Using a line tool of ZEN software, we marked positive cells at the single fluorescent image for AlexaFluor488 or AlexaFluor594, separately and subsequently classified positive cells into 5-HT positive, tdTomato positive and 5-HT/tdTomato positive neurons.

### High-performance liquid chromatography (HPLC)

After cervical dislocation, the brain was immediately removed and sectioned coronary at 2.4 mm posterior from bregma. The anterior part of the brain was placed on ice and processed for HPLC analysis, whereas the posterior part of the brain was used for immunohistochemistry to assess the efficiency of the cell ablation. The brain was homogenized with 0.2 M perchloric acid, 100 μM EDTA2·Na in MilliQ (0.5 mL/ 100 mg brain) and 200 ng isoproterenol as an internal standard.

The homogenized sample was left on ice for 30 min and then centrifuged at 20,000 × *g* for 15 min at 0°C. The pH of the supernatant was adjusted to be approximately pH 3, and the supernatant was then filtered through a 0.45 μm Millex filter (Millipore). The noradrenalin, 5-HT, 5-HIAA, dopamine, DOPAC, and HVA in the solutions were separated using an Eicompak SC-5ODS column (Eicom) and subsequently detected using an electrochemical detector HTEC-500 (Eicom). The concentration was calculated based on peak areas which were quantified based on the external standerd calibration employing linear regression analysis using LC solution software (Shimadzu). Norepinephrine bitartratesalt (Sigma N5785), Serotonin creatininesulfate (Sigma H7752), 5-Hydroxyindoleaceticacid (Sigma H8876), Dopamin hydrochloride (Sigma H8502), 3,4-Dihydroxyphenylacetic acid (Sigma 850217), and Homovanillicacid (Sigma H1252) dissolved in 0.1 M acetic acid, 50 μM EDTA2·Na in MilliQ were measured as external standerds in each experiment.

## Statistical analysis

No method of randomization was used in any of the experiments. The experimenters who staged sleep/wakefulness based on EEG/EMG were blinded to treatment assignment. Statistical analysis was performed using SPSS Statistics 22 (IBM). All of the data were tested for Gaussian distribution and variance. For group comparisons among the control group and the groups treated with 2.5 ng and 5 ng DT, the number of neurons was analyzed using one-way ANOVA followed by *post hoc* Tukey's test. Wake response was analyzed using two-way ANOVA followed by post hoc Tukey's test. To compare the parameters of paired groups, we performed paired *t*-test with Bonferroni correction. We performed Wilcoxon matched-pairs signed-rank test with Bonferroni correction to compare the parameters of paired groups when the data did not follow Gaussian distribution.

## Results

### Ablation of central 5-HT neurons in adult mice

**Figure 1:**
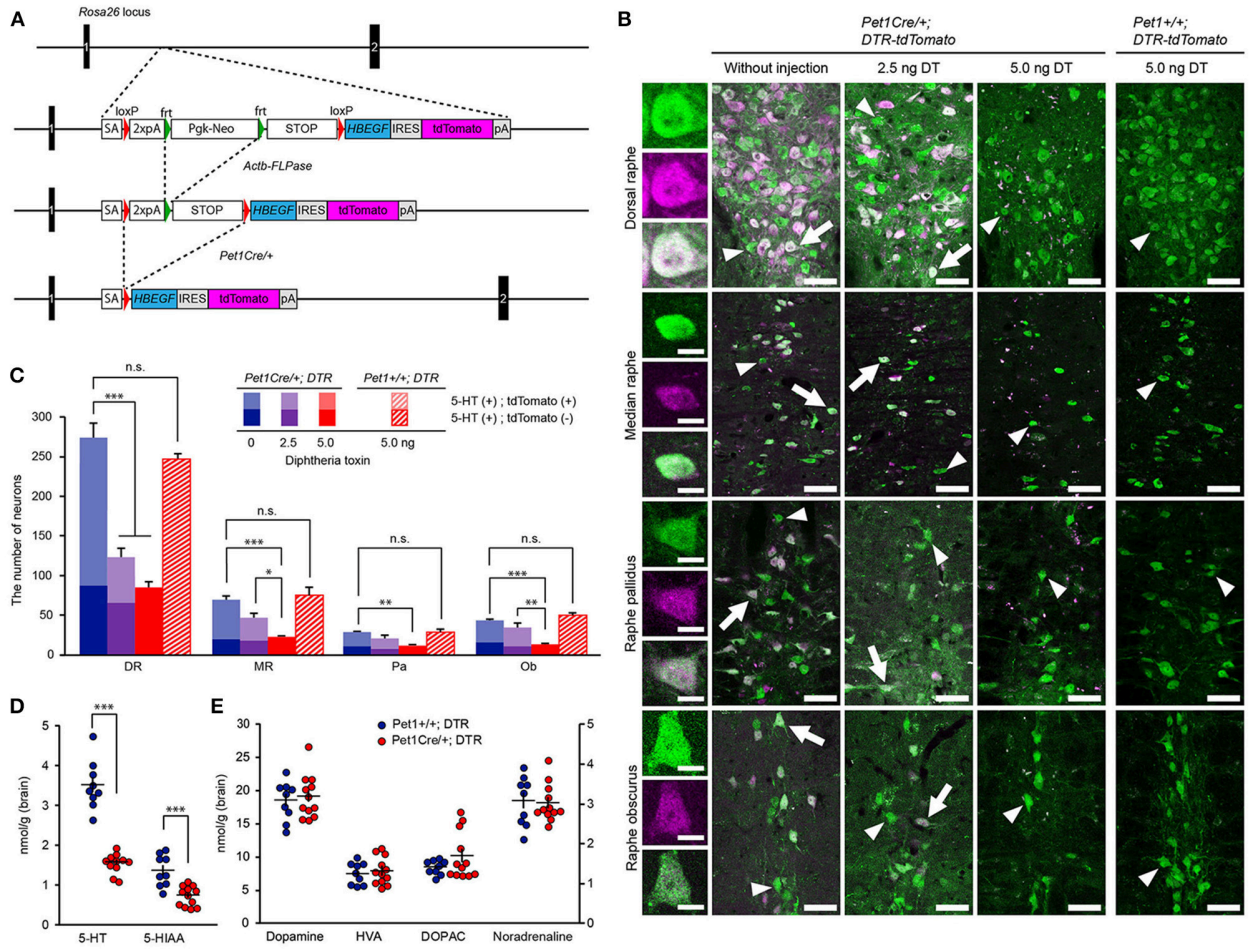
Ablation of central 5-HT neurons. **(A)** Schematic diagram of the wild-type (WT) Rosa26 locus, PGK-Neo-STOP- HBEGF-tdTomato allele, PGK-Neo deleted by β*-actin*^*FLPase*/+^ and the allele activated by Pet1-Cre-mediated deletion of the STOP sequence. **(B)** Leftmost column: Representative images of cells double positive for 5-HT (green) and tdTomato (magenta) with a merged image (white). Second column: The raphe nuclei contained many cells that were doubly positive for tdTomato and 5-HT cells in *Pet1*^*Cre*/+^; *Rosa-DTR-tdTomato* mice without diphtheria toxin (DT) administration. Third column: There were many remaining cells that were doubly positive for tdTomato and 5-HT after the administration of 2.5 ng DT. Fourth column: After the administration of 5 ng DT, no tdTomato-positive cells were observed in the raphe nuclei. Arrows indicate cells doubly positive for 5-HT and tdTomato. Arrowheads indicate 5-HT-positive, tdTomato-negative cells. Rightmost column: After the administration of 5 ng DT, the raphe nuclei contained many 5-HT cells in *Pet1*^+/+^; *Rosa-DTR-tdTomato* mice. Scale bars: 10 μm for the leftmost column, 50 μm for other columns. **(C)** The number of serotonergic neurons in raphe nuclei without (*n* = 4) and after the administration of 2.5 ng (*n* = 3–6) and 5 ng DT (*n* = 5) in *Pet1*^*Cre*/+^; *Rosa-DTR-tdTomato* mice, and 5 ng DT (*n* = 3–4) in *Pet1*^+/+^; *Rosa-DTR-tdTomato* mice. One-way ANOVA followed by Tukey's test. **(D,E)** Quantification of 5-HT, 5-HIAA, dopamine, HVA, DOPAC, and noradrenaline in brain homogenates of DT-administered *Pet1*^*Cre*/+^; *Rosa-DTR-tdTomato* (*n* = 12) and *Pet1*^+/+^; *Rosa-DTR-tdTomato* mice (*n* = 9). Two-tailed *t*-test. *p < 0.05, **p < 0.01, ***p < 0.001. The data are presented as the group mean ± S.E.M.

Whereas simian or human are sensitive to DT, mice are resistant to DT (Pappenheimer et al., 1982; Saito et al., 2001). The species difference in sensitivity to DT depends on the binding affinity of DT to heparin-binding epidermal growth factor (HBEGF). Simian and human HBEGF works as the receptor for DT (DTR) (Mitamura et al., 1995). To render a specific group of neurons sensitive to DT in a Cre-dependent manner, we generated mice in which the Rosa26 locus was modified by a targeted insertion of a construct that contained loxP-Stop-loxP-simian HBEGF or DTR, followed by internal ribosomal entry site (IRES)-tdTomato (Figure 1A). In these mice, DTR is expressed in the presence of Cre protein in cells that can be visualized by a fluorescent protein, tdTomato. *Rosa26-DTR-tdTomato* mice were crossed with *Pet1*^*Cre*^ mice (Scott et al., 2005; Liu et al., 2010), in which Cre is expressed in 5-HT neurons, intestinal epithelial cells and pancreatic islet cells in adult mice (Scott et al., 2005). In the pons of *Pet1*^*Cre*^*; Rosa26-DTR-tdTomato* mice, the percentage of 5-HT-postive cells that were positive for tdTomato was 68.1 ± 2.7 (mean ± SEM) % and 71.2 ± 4.1% in the dorsal and median raphe, respectively (Figures 1B,C), whereas 4.4 ± 0.8% and 12.6 ± 1.2% of tdTomato-positive cells were negative for 5-HT in the dorsal and median raphe, respectively. In the medulla, the percentage of 5-HT-postive cells that were positive for tdTomato was 63.6 ± 5.1% and 64.1 ± 3.1% in the raphe pallidus and raphe obscurus, respectively, whereas 9.9 ± 2.8% and 11.9 ± 1.9% of tdTomato-positive cells were negative for 5-HT in the raphe pallidus and raphe obscurus, respectively, which is consistent with the results of a previous study (Cerpa et al., 2014) (Figures 1B,C).

First, we determined the amount of DT necessary to ablate all tdTomato-positive cells expressing DTR. Since DT ablated the neurons within 1 week (Wu et al., 2008), we quantified the remaining tdTomato-positive cells 12 days after ICV DT administration. When 2.5 ng of DT was administered, many tdTomato-positive cells survived in the raphe nuclei of the *Pet1*^*Cre*/+^; *Rosa-DTR-tdTomato* mice (Figures 1B,C). However, 5 ng of DT completely ablated the tdTomato-positive cells in the raphe nuclei (Figures 1B,C). After the administration of 5 ng of DT, the number of 5-HT neurons was decreased by 69% in the dorsal raphe compared with that in the non-injected mice. The number of 5-HT neurons in the median raphe, raphe pallidus and raphe obscurus decreased to 33, 35, and 30%, respectively, compared with that in the non-injected mice (Figures 1B,C), consistent with the percentage of all 5-HT neurons negative for tdTomato (Figures 1B,C). The administration of 10 ng of DT did not decrease the number of remaining 5-HT neurons (data not shown). The administration of 5 ng DT did not ablate 5-HT neurons of *Pet1*^+/+^; *Rosa-DTR-tdTomato* mice in which no DTR was observed (Figure 1C). At 9 days after the administration of 5 ng of DT, the number of central 5-HT neurons was significantly lower than that in control mice and similar to that observed 12 days after the DT administration (101.0 ± 4.6 cells in the dorsal raphe, *P* = 0.71 vs. the 12th day; 34.0 ± 0.8 cells in the median raphe, *P* = 0.11 vs. the 12th day; 11.0 ± 1.7 cells in the raphe pallidus, *P* = 0.82 vs. the 12th day; 14.7 ± 1.5 in the raphe obscurus, *P* = 0.66 vs. the 12th day, *n* = 3, one-way ANOVA with Tukey's *post hoc* test). Thus, we used 5 ng of DT to assess the effect of central 5-HT neurons on sleep/wake behaviors.

We confirmed the reduction in 5-HT levels after the ablation of 5-HT neurons using high-performance liquid chromatography (HPLC) of forebrain homogenates. DT-administered *Pet1*^*Cre*/+^; *Rosa-DTR-tdTomato* mice showed that a 55.3 and 46.1% reduction in 5-HT and its metabolite, 5-hydroxyindoleacetic acid (5-HIAA), respectively, compared with DT-administered *Pet1*^+/+^; *Rosa-DTR-tdTomato* mice (Figure 1D). To further examine whether central 5-HT neuron ablation affected the amount of other monoamines, we quantified the levels of noradrenaline, dopamine, and dopamine metabolites 3,4-dihydroxyphenylacetic acid (DOPAC) and homovanillic acid (HVA) in forebrain homogenates, which were comparable between forebrain homogenates from DT-administered *Pet1*^*Cre*/+^; *Rosa-DTR-tdTomato* and DT-administered *Pet1*^+/+^; *Rosa-DTR-tdTomato* mice (Figure 1E).

### Physical parameters after central 5-HT neuron ablation

**Figure 2:**
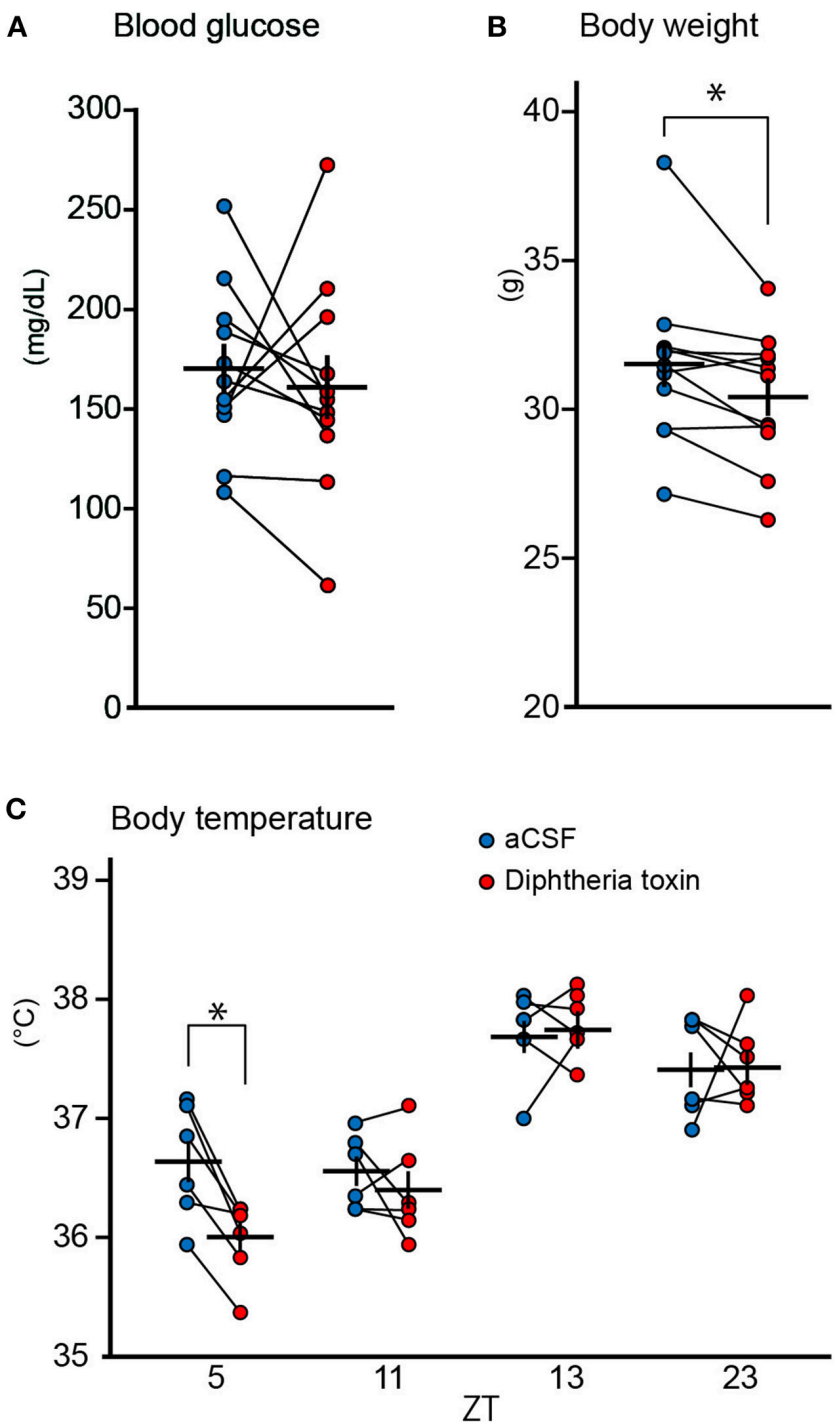
Physical parameters of central 5-HT neuron-ablated mice. **(A)** Blood glucose of *Pet1*^*Cre*/+^; *Rosa-DTR-tdTomato* mice before and after the administration of 5 ng (*n* = 11) diphtheria toxin (DT). **(B)** Body weight of *Pet1*^*Cre*/+^; *Rosa-DTR-tdTomato* mice (*n* = 11) before and after the administration of 5 ng DT. **(C)** Body temperature of *Pet1*^*Cre*/+^; *Rosa-DTR-tdTomato* mice (*n* = 6) after the administration of 5 ng DT and aCSF. Paired two-tailed *t*-test with Bonferroni correction. *p < 0.05. The data are presented as the group mean ± S.E.M.

Since central 5-HT neurons have been reported to be involved in glucose metabolism and body temperature (Cerpa et al., 2014; McGlashon et al., 2015), we examined whether DT administration affected the serum glucose level, body temperature, and body weight. Although 5 ng DT did not affect the serum glucose level (Figure 2A), slightly but significantly reduced body weight [change in body weight; −1.1 ± 0.37 g (mean ± S.E.M), Figure 2B]. We measured body temperature of *Pet1*^*Cre*/+^; *Rosa-DTR-tdTomato* mice at ZT5, 11, 13, and 23 on the 9th and 10th days after the DT administration. Mice are most often asleep at ZT5 and are awake and most active at ZT13. ZT11 and ZT23 are 1 h before the light-off and light-on, respectively. DT administration did not alter body temperature at ZT11, 13 and 23 (change in body temperature; −0.15 ± 0.15°C at ZT11, 0.08 ± 0.19°C at ZT13, 0.03 ± 0.21°C at ZT23) but decreased at ZT5 (change in body temperature; −0.63 ± 0.12°C, Figure 2C).

### Decreased REM sleep in central 5-HT neuron-ablated mice

**Figure 3:**
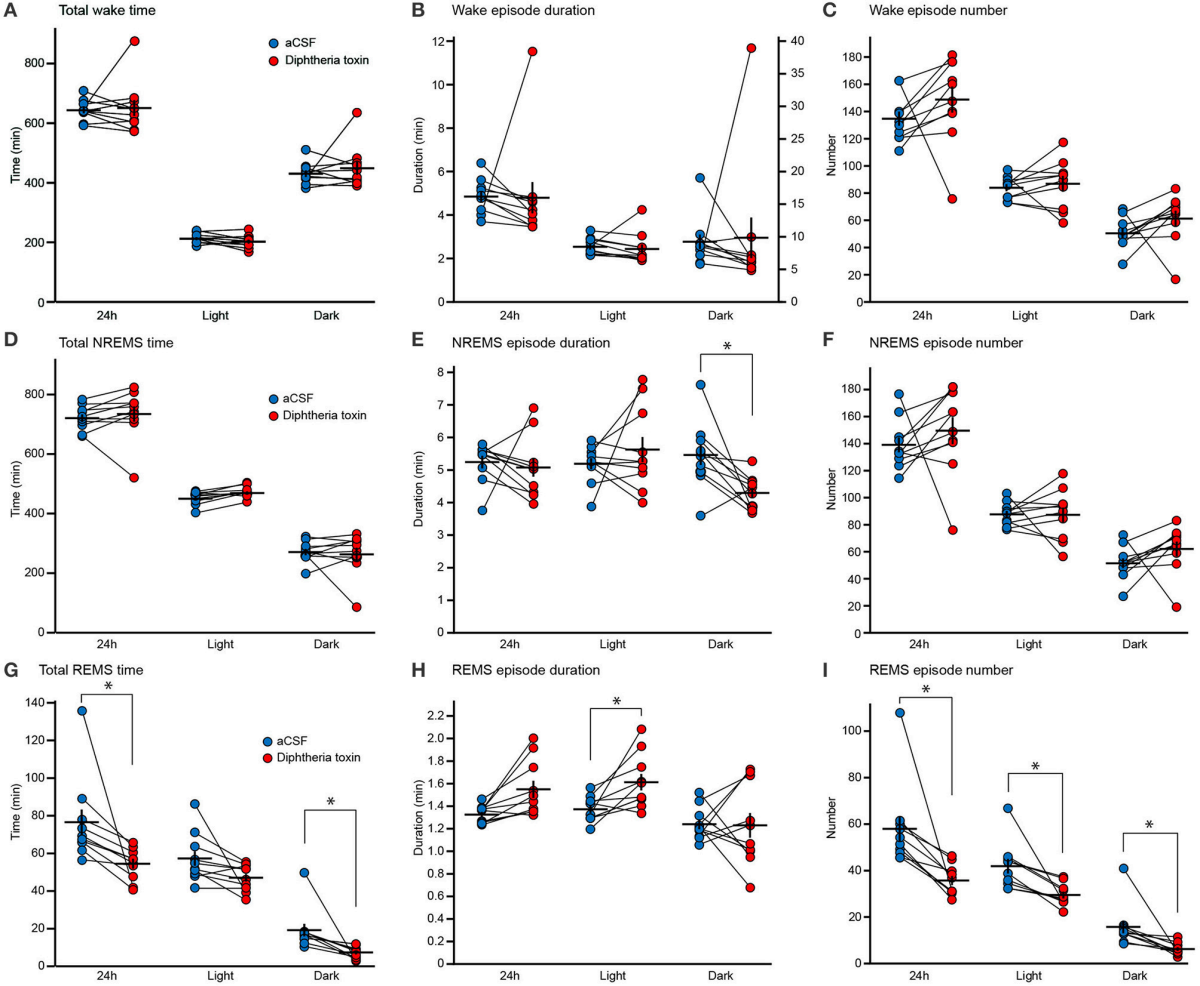
Sleep/wake behaviors of central 5-HT neuron-ablated mice. **(A–C)** Total time spent in wakefulness **(A)**, wake episode duration **(B)** and wake episode number **(C)** of aCSF- and diphtheria toxin (DT)-administered *Pet1*^*Cre*/+^; *Rosa-DTR-tdTomato* mice. **(D–F)** Total time spent in NREM sleep (NREMS) **(D)**, NREMS episode duration **(E)** and NREMS episode number **(F)** of aCSF- and DT-administered *Pet1*^*Cre*/+^; *Rosa-DTR-tdTomato* mice. **(G–I)** Total time spent in REM sleep (REMS) **(G)**, REMS episode duration **(H)** and REMS episode number **(I)** of aCSF- and DT-administered *Pet1*^*Cre*/+^; *Rosa-DTR-tdTomato* mice. Ten mice in each group. *p < 0.05. Wilcoxon matched-pairs signed-rank test with Bonferroni correction. The data are presented as the group mean ± S.E.M.

**Figure 4:**
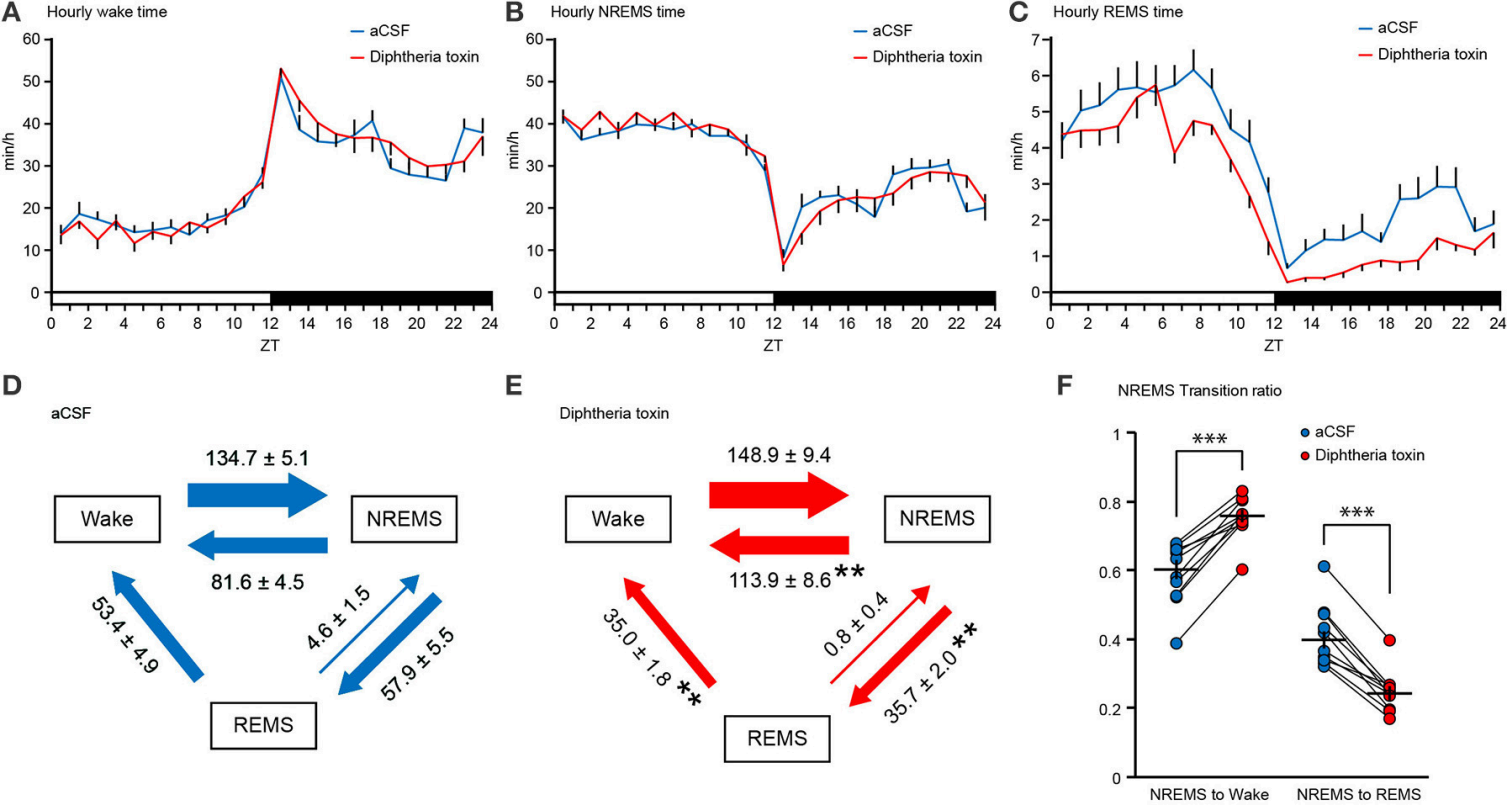
Daily variation and state transitions of central 5-HT neuron-ablated mice. **(A–C)** Daily variation in wakefulness **(A)**, NREM sleep **(B)** and REM sleep **(C)** in aCSF- and diphtheria toxin (DT)-administered *Pet1*^*Cre*/+^; *Rosa-DTR-tdTomato* mice. The values indicate minutes per hour spent in each stage, averaged from EEG/EMG recordings during two consecutive 24-h periods. **(D,E)** The values indicate the number of transitions between wakefulness, NREM sleep, and REM sleep per 24 h in aCSF **(D)**- and DT **(E)**-administered *Pet1*^*Cre*/+^; *Rosa-DTR-tdTomato* mice. **(F**) The graph indicates transition ratios from NREM sleep to wake and from NREM sleep to REM sleep. Ten mice in each group. **p < 0.01, ***p < 0.001. Paired two-tailed *t*-test. The data are presented as the group mean ± S.E.M.

We assessed the sleep/wake behaviors of DT-administered mice 9 and 10 days after DT administration. Central 5-HT neuron ablation did not alter the time spent in wakefulness (Figure 3A), the wake episode duration (Figure 3B) or the wake episode number (Figure 3C). Furthermore, the daily variation in wake time was similar between artificial cerebrospinal fluid (aCSF)- and DT-administered *Pet1*^*Cre*/+^; *Rosa-DTR-tdTomato* mice (Figure 4A).

DT-administered *Pet1*^*Cre*/+^; *Rosa-DTR-tdTomato* mice did not alter total NREM sleep time (Figure 3D). The ablation of central 5-HT neurons decreased the NREM sleep episode duration during the dark phase (Figure 3E). There was no significant difference in the number of NREM sleep episodes (Figure 3F), and the daily variation in NREM sleep time was similar between aCSF- and DT-administered *Pet1*^*Cre*/+^; *Rosa-DTR-tdTomato* mice (Figure 4B).

The ablation of central 5-HT neurons decreased the time spent in REM sleep by approximately 24.7% daily and 53.6% in the dark phase but the difference did not reach significance during the light phase (Figures 3G, 4C). Furthermore, central 5-HT neuron ablation increased the duration of REM sleep episodes during the light phase (Figure 3H). Finally, DT-administered *Pet1*^*Cre*/+^; *Rosa-DTR-tdTomato* mice exhibited fewer REM sleep episodes during both the light and dark phases than those administered aCSF (Figure 3I).

The ablation of central 5-HT neurons decreased the transitions from NREM sleep to REM sleep by 34.7% (Figures 4D,E). The number of transitions from REM sleep to wakefulness were also decreased in DT-administered *Pet1*^*Cre*/+^; *Rosa-DTR-tdTomato* mice compared with that in mice given aCSF. Conversely, the transitions from NREM sleep to wakefulness increased in central 5-HT neuron-ablated mice (Figures 4D,E). To normalize the difference in NREM sleep episode number of each mouse, we calculated the transition ratio of NREM sleep episodes. DT-administered *Pet1*^*Cre*/+^; *Rosa-DTR-tdTomato* mice showed a higher transition ratio from NREM sleep to wake and a lower transition ratio from NREM sleep to REM sleep than aCSF-administered *Pet1*^*Cre*/+^; *Rosa-DTR-tdTomato* mice (Figure 4F).

### Increased theta power during wakefulness of central 5-HT neuron-ablated mice

**Figure 5:**
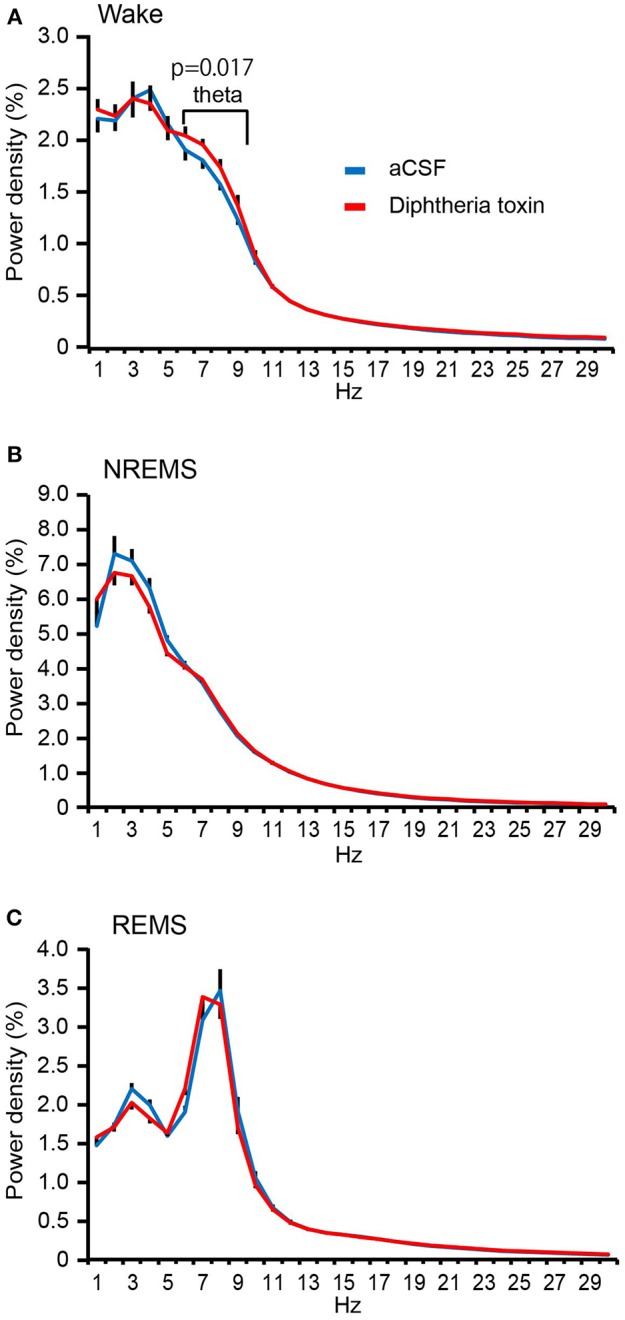
EEG spectrum analysis of central 5-HT neuron-ablated mice. **(A)** Power density during wakefulness in aCSF- and diphtheria toxin (DT)-administered *Pet1*^*Cre*/+^; *Rosa-DTR-tdTomato* mice. **(B)** Power density during NREM sleep in aCSF- and DT-administered *Pet1*^*Cre*/+^; *Rosa-DTR-tdTomato* mice. **(C)** Power density during REM sleep in aCSF- and DT-administered *Pet1*^*Cre*/+^; *Rosa-DTR-tdTomato* mice. Ten mice in each group. Paired two-tailed *t*-test for delta and theta range density. The data are presented as the group mean ± S.E.M.

The EEG spectral analysis of the wake state revealed that the theta (6–9 Hz)-range power density in *Pet1*^*Cre*/+^; *Rosa-DTR-tdTomato* mice given DT was significantly higher than that in aCSF-administered *Pet1*^*Cre*/+^; *Rosa-DTR-tdTomato* mice (Figure 5A) without any significant change in the power density in the delta (1–4 Hz) range. Furthermore, the ablation of central 5-HT neurons did not alter the EEG spectrum during NREM sleep (theta range, *P* = 0.588; delta range, *P* = 0.524; Figure 5B) or REM sleep (theta range, *P* = 0.818; delta range, *P* = 0.270; Figure 5C).

### Ablation of central 5-HT neurons attenuated the arousal response to a novel environment

**Figure 6:**
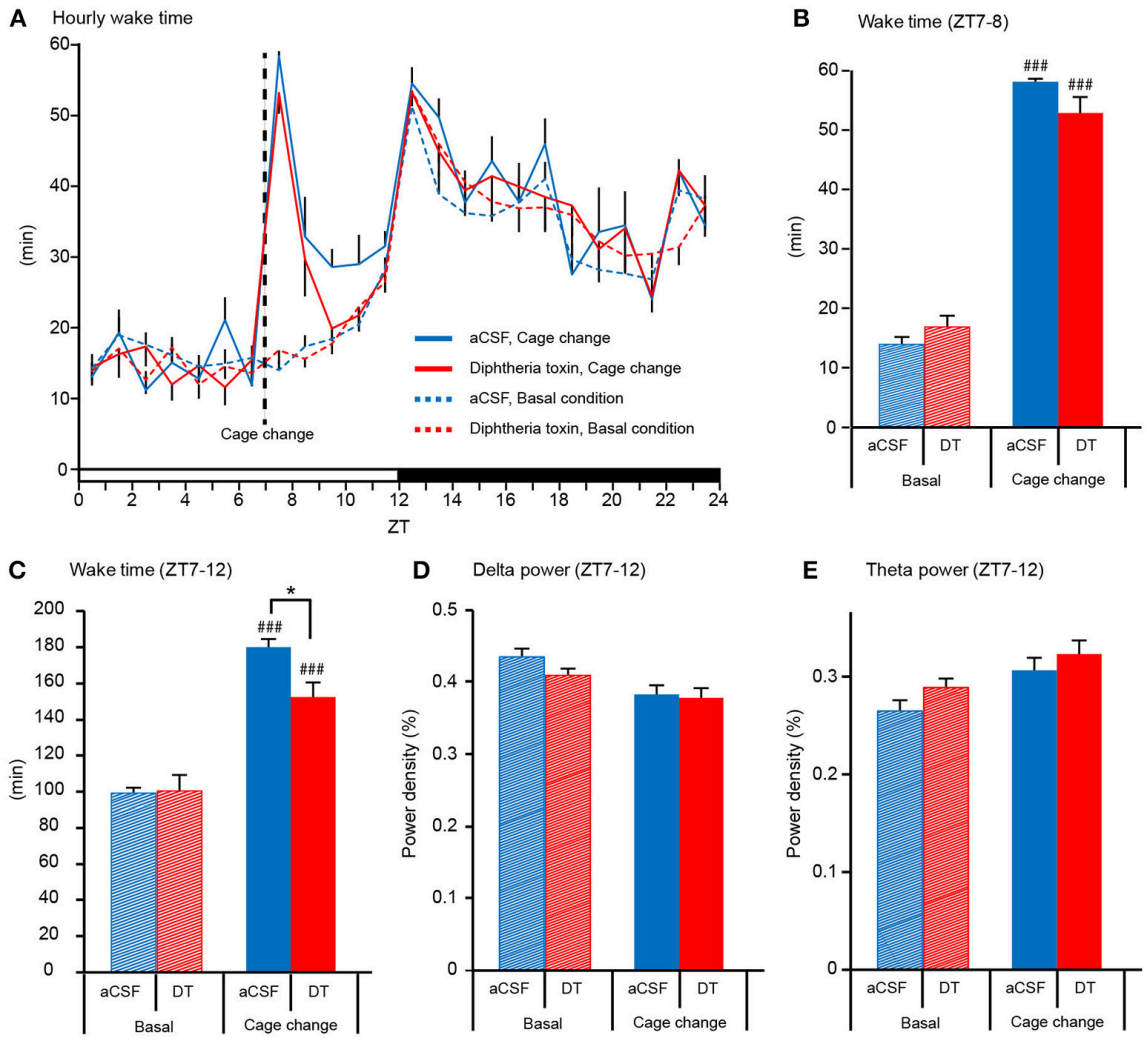
Arousal response of central 5-HT neuron-ablated mice. **(A)** Hourly time spent in wakefulness before and after the mouse was transferred into a novel cage at ZT7. **(B)** Time spent in wakefulness of aCSF- and diphtheria toxin (DT)-administered *Pet1*^*Cre*/+^; *Rosa-DTR-tdTomato* mice for 1 h beginning at ZT7. **(C)** Time spent in wakefulness of aCSF- and DT-administered *Pet1*^*Cre*/+^; *Rosa-DTR-tdTomato* mice for 5 h from ZT7 to ZT12. Basal condition, *n* = 10. Cage change, *n* = 8. **(D)** Delta and **(E)** theta power density during wakefulness from ZT7 to ZT12. Six mice in each group. *p < 0.05. ###*p* < 0.001, vs. basal groups. Two-way ANOVA followed by Tukey's test. The data are presented as the group mean ± S.E.M.

To further examine the wake-promoting effect of 5-HT neurons, we assessed the sleep/wake behavior after the mice were moved into a novel cage during the light phase, as previously performed with mice deficient in wake-promoting transmitters such as histamine, noradrenaline, and orexin (Parmentier et al., 2002; Hunsley and Palmiter, 2003; Mochizuki et al., 2004). Central 5-HT neuron-ablated mice exhibited a strong arousal response similar to control mice during the first 1 h after the cage change at ZT7 (Figures 6A,B), but the time spent in wakefulness of 5-HT-ablated mice was significantly less than that of aCSF-administered mice during the remaining light phase from ZT7 to ZT12 (Figures 6A,C), suggesting a shorter duration of the arousal response in DT-administered *Pet1*^*Cre*/+^; *Rosa-DTR-tdTomato* mice. Although DT-administered *Pet1*^*Cre*/+^; *Rosa-DTR-tdTomato* mice showed shorter total wake time during the light phase after cage changes than aCSF-administered *Pet1*^*Cre*/+^; *Rosa-DTR-tdTomato* mice, both delta power and theta power during wakefulness from ZT7 to ZT12 were similar between the two groups (Figures 6D,E).

## Discussion

The present study demonstrated that the ablation of central 5-HT neurons in adult mice resulted in a reduction in total REM sleep time that was associated with fewer transitions from NREM sleep to REM sleep. Central 5-HT neuron-ablated mice also showed an attenuated arousal response to a novel environment compared to control mice.

DT kills cells by inhibiting protein synthesis through ADP-ribosylation of elongation factor 2 (Murphy, 2011). Since the amount of DT that is required to ablate target cells differs depending on the route of administration and the location of the target cells, we need to determine the minimum amount of DT that reliably ablates all cells expressing DTR. The *Rosa-DTR-tdTomato* mouse is a useful mouse line in which the tdTomato protein allows for the identification of cells susceptible to DT and examination of the efficiency of DT-induced cell ablation.

Pet1 is necessary for the proper differentiation and function of almost all 5-HT neurons (Wyler et al., 2015, 2016). Although *Pet1*-deficient mice have 20–30% of 5-HT neurons compared with wild-type mice (Kiyasova et al., 2011), the remaining 5-HT neurons in *Pet1*-deficient mice lack the expression of genes such as Tph2, serotonin transporter, organic cation transporter 3 (Slc22a3), and alpha 1 adrenergic receptor that are required for the differentiation and function of 5-HT neurons (Wyler et al., 2015, 2016). The presence of tdTomato-negative, 5-HT-positive cells in *Pet1*^*Cre*/+^*; Rosa-DTR-tdTomato* mice was consistent with a previous study using *Pet1*^*Cre*/+^ mice (Cerpa et al., 2014) and may be caused by imperfect recombination efficiency, which was suggested by the comparison between Sert-Cre and Pet1-Cre activity on a floxed vesicular monoamine transporter 2 gene (Narboux-Nême et al., 2011, 2013).

The current study showed decreased REM sleep after central 5-HT neuron ablation. Consistent with this finding, *Pet1-Cre; Lmx1b*^*flox*/*flox*^ mice that did not produce central 5-HT exhibited less REM sleep than wild-type mice (Buchanan and Richerson, 2010). Since mice deficient in Gs-coupled 5-HT7 exhibited a decrease in the total time of REM sleep (Hedlund et al., 2005) but no change was observed in mice deficient in Gq-coupled 5-HT2A (Popa et al., 2005) and 5-HT2C (Frank et al., 2002), the loss in 5-HT7/Gs-signaling may be crucial for the observed reduction in REM sleep time after central 5-HT neuron ablation. The increased total REM sleep time in mice deficient in the Gi-coupled autoreceptors 5-HT1A (Boutrel et al., 2002) and 5-HT1B (Boutrel et al., 1999) may be explained by enhanced 5-HT7/Gs signaling subsequent to the loss of inhibitory autoreceptors. 5-HT neurons are regarded as “REM-off” neurons, which cease firing during REM sleep (McGinty and Harper, 1976; Trulson and Jacobs, 1979; Jacobs and Fornal, 1999). The observation that the loss of “REM-off” 5-HT neurons decreases REM sleep time is counterintuitive. One potential explanation for these confounding observation is that the activity of 5-HT neurons during NREM sleep enhances the tendency for a state transition from NREM sleep toward REM sleep and away from wakefulness, which could explain the reduction in the transition from NREM sleep to REM sleep after central 5-HT neuron ablation observed in the present study.

5-HT neuron-ablated mice exhibited an attenuated arousal response to a novel environment compared to the control mice, which has been reported in mice deficient in histidine decarboxylase, a rate-limiting enzyme for the synthesis of histamine (Parmentier et al., 2002), and in mice deficient in dopamine β-hydroxylase (Hunsley and Palmiter, 2003). This finding supports the role of 5-HT neurons as wake-promoting neurons, consistent with the findings that optogenetic activation of 5-HT neurons in the dorsal raphe enhanced wakefulness (Ito et al., 2013), activation of 5-HT neurons using the designer receptor exclusively activated by designer drugs (DREADD) system increased regional cerebral blood flow in many cortical and subcortical areas including the ventral tegmental area (Giorgi et al., 2017), and 5-HT neurons are usually more active during wakefulness (McGinty and Harper, 1976; Trulson and Jacobs, 1979; Jacobs and Fornal, 1999). However, video monitoring has shown a decrease in sleep time in adult 5-HT deficiency (Whitney et al., 2016), suggesting that the effect of 5-HT neuron ablation on sleep/wakefulness could be different from that of adult 5-HT deficiency, potentially because glutamate transmission from 5-HT neurons may contribute to wakefulness (Fu et al., 2010; Hioki et al., 2010; Liu et al., 2014).

Although the current study failed to detect any changes in daily total wake time after the ablation of 5-HT neurons, this result is consistent with previous studies on mice that were deficient in wake-promoting neurotransmitters, such as histamine, noradrenalin and orexin, which showed normal total wake time under basal conditions (Parmentier et al., 2002; Hunsley and Palmiter, 2003; Mochizuki et al., 2004). Since multiple wake-promoting circuits work together to maintain baseline wakefulness, the redundancy in the wake-promoting system may be able to compensate for the loss of 5-HT neurons in basal wakefulness but may not be sufficient for a full arousal response in a novel cage.

The current study also showed higher theta power during wakefulness of central 5-HT neuron-ablated mice, suggesting a suppressing effect of 5-HT signaling in hippocampal theta generation. The theta power during wakefulness usually increases during exploratory behaviors and may play a role in navigation (Buzsáki, 2005; Bender et al., 2015). Thus, our results indicates 5-HT neurons may negatively regulate hippocampal theta rhythm during navigation and locomotive behavior. Consistently, systemic administration of selective agonists for autoreceptors 5-HT1A and 1C enhanced the hippocampal theta of freely moving animals (Marrosu et al., 1996; Sörman et al., 2011). In addition, 5-HT fibers were found to be abundant in the hippocampus of wild-type mice but drastically decreased in the hippocampus of *Pet1*-deficient mice (Kiyasova et al., 2011). Although the theta power of central 5-HT neuron-ablated mice from ZT7-12 did not reach statistical significance (Figure 6E), this may be due to a smaller number of mice examined.

A limitation of the current study was that the role of 5-HT neurons in sleep/wake behavior could be underestimated due to the effect of the remaining 5-HT neurons after the ablation. Total loss of central 5-HT neurons may cause a further decrease in total REM sleep time and a weaker arousal response compared to the current results, and could result in an decrease in total wake time and a increase in total NREM sleep time. The number of 5-HT neurons was decreased by 68%, and the 5-HT content was decreased by 55%. The smaller reduction in 5-HT content than in 5-HT neuron number can be partly explained by 5-HT derived from the blood. Tph2-deficient mice have 4–7% of the 5-HT found in control mice in the brain or cerebral cortex (Savelieva et al., 2008; Alenina et al., 2009). A reduction in negative feedback via autoreceptor 5-HT1a may also work to increase 5-HT synthesis by the residual 5-HT neurons.

The raphe pallidus neurons directly connect to the sympathetic preganglionic neurons which activate the brown adipose tissue to enhance heat production (Nakamura, 2011). Thus, ablation of the raphe pallidus neuron may account for mild reduction in body temperature. The ablation of central 5-HT neurons did not reduce body temperature at ZT11, 13, and 23 but decreased at ZT5 by 0.6°C, suggesting that central 5-HT neuron ablation resulted in a decrease in body temperature during the early-mid light phase. Since energy expenditure that is associated with locomotion and thermic effect of food is much higher during the dark phase, than during the light phase (Abreu-Vieira et al., 2015). Therefore, we think that low body temperature of central 5-HT neuron-ablated mice during the light phase suggests that preserved energy expenditure associated with locomotion and/or food digestion. It is also possible that central 5-HT neuron ablation disturbs circadian change in body temperature which is regulated by the suprachiasmatic nucleus where several 5-HT receptors are expressed (Versteeg et al., 2015). Compared with our results, larger reduction in body temperature was reported on 5-HT neuron-ablated Pet1/DTR mice through intraperitoneal administration of diphtheria toxin (Cerpa et al., 2014; McGlashon et al., 2015). However, systemic administration of diphtheria toxin ablated many Pet1-positive cells, including pancreatic islet cells (Ohta et al., 2011), which results in a severe diabetic condition (Jia et al., 2014) that is usually accompanied by hypothermia. Consistently, Tph2-deficient mice showed a body temperature that was similar to wild-type mice (Solarewicz et al., 2015).

Since central 5-HT neuron ablation decreased total REM sleep time and NREM sleep episode duration during the dark phase but not during the light phase, it is unlikely that the reduction in body temperature caused a decrease in REM sleep time and NREM sleep duration in the central 5-HT neuron-ablated mice. However, we cannot deny the possibility that this mild reduction in body temperature during the light phase affects the transition frequency from NREM sleep to REM sleep and the arousal response in response to a novel cage. Furthermore, the sleep/wake state and body temperature affect each other (Murray et al., 2015), making a simple conclusion difficult to draw. If distinct subgroups of 5-HT neurons separately regulate sleep/wakefulness and body temperature, a future study manipulating specific projections of 5-HT neurons may elucidate this issue by separating the behavioral effects of central 5-HT neuron ablation. Central 5-HT neuron ablation tends to decrease both body temperature and body weight. Given that decreased body temperature is closely correlated with reduced energy expenditure, central 5-HT neuron-ablated mice may show a reduction in food intake. Further study is needed to examine the role of central 5-HT neurons in energy metabolism including food intake and oxygen consumption.

In summary, the current study demonstrates that the *Rosa-DTR-tdTomato* mouse is a useful mouse line in which the ablation efficiency of target cells is easily evaluated and suggests a crucial role of central 5-HT neurons in regulating REM sleep time, the transition from NREM sleep to REM sleep and the arousal response.

## Data availability

All data are available upon request from the corresponding author.

## Author contributions

HF and MY conceived and designed the experiments. KI, HK, MK and CM performed the experiments. KI and HF analyzed the data. KS and MA contributed reagents, materials, analysis tools. KI, HF and MY wrote the paper.

### Conflict of interest statement

The authors declare that the research was conducted in the absence of any commercial or financial relationships that could be construed as a potential conflict of interest.
